# Antibodies to actin in autoimmune haemolytic anaemia

**DOI:** 10.1186/1746-6148-6-18

**Published:** 2010-03-30

**Authors:** Kathrin M Felder, Katharina Hoelzle, Karl Heinritzi, Mathias Ritzmann, Ludwig E Hoelzle

**Affiliations:** 1 Institute of Veterinary Bacteriology, Vetsuisse Faculty, University of Zurich, Switzerland; 2 Clinic for Swine, Ludwig-Maximilians-University Munich, Munich, Germany; 3 Clinic for Swine, University for Veterinary Medicine, Vienna, Austria

## Abstract

**Background:**

In autoimmune haemolytic anaemia (AIHA), autoreactive antibodies directed against red blood cells are up-regulated, leading to erythrocyte death. *Mycoplasma suis *infections in pigs induce AIHA of both the warm and cold types. The aim of this study was to identify the target autoantigens of warm autoreactive IgG antibodies. Sera from experimentally *M. suis*-infected pigs were screened for autoreactivity.

**Results:**

Actin-reactive antibodies were found in the sera of 95% of all animals tested. The reactivity was species-specific, i.e. reactivity with porcine actin was significantly higher than with rabbit actin. Sera of animals previously immunised with the *M. suis *adhesion protein MSG1 showed reactivity with actin prior to infection with *M. suis *indicating that molecular mimicry is involved in the specific autoreactive mechanism. A potentially cross-reactive epitope was detected.

**Conclusions:**

This is the first report of autoreactive anti-actin antibodies involved in the pathogenesis of autoimmune haemolytic anaemia.

## Background

Haemotrophic mycoplasma infections are found in a wide range of domestic and wild animals causing acute haemolytic anaemia or chronic anaemia with immune suppression [1,2]. Generally, they show distinct host specificity. However, there are reports of humans infected with haemotrophic mycoplasmas, i.e. *Mycoplasma haemofelis *and *Mycoplasma suis *[3,4]. *M. suis *causes infectious anaemia in pigs (IAP) [1].

Two forms of autoimmune haemolytic anaemia (AIHA) have been described: warm AIHA, which is characterised by autoreactive IgG antibodies binding their target epitope at body temperature [5], and cold AIHA. In cold AIHA, complement-activating autoreactive IgM antibodies bind their antigen, usually the l/i epitope on red blood cells, below body temperature and are strongly agglutinating at 4°C [6,7]. Both types of antibodies occur in 7% of human AIHA cases; this is referred to as mixed AIHA. Sokol and co-workers report only one case of secondary mixed AIHA due to *Mycoplasma *infection, i.e. *M. pneumoniae *[8].

Mixed AIHA has been found in *M. suis*-induced IAP. Cold IgM agglutinins targeting glycoproteins on the red blood cells were observed [9,10]. These cold agglutinins occur about four weeks after experimental infection with *M. suis*. During the acute stage of IAP (clinical attack), acute anaemia, hypoglycaemia and icteroanaemia accompanied by high mortality occur. It is assumed that the severe anaemia observed in pigs suffering from acute clinical signs is due to a combination of direct damage to the red blood cells by *M. suis *attachment and invasion, and upregulation of cold and warm autoreactive antibodies directed against red blood cell components. In previous studies, these autoreactive warm IgG antibodies have been shown to overwhelm the specific immune response to *M. suis *[11].

In this study, sera from experimentally *M. suis*-infected pigs were screened for the presence of warm IgG antibodies by testing their reactivity with blood preparations from healthy pigs. The detection and characterisation of the host proteins acting as autoantigens was a main issue. Earlier studies, i.e. serological proteome analysis [12], yielded some evidence that actin is a target protein for the autoreactive antibodies detected during the acute state of the disease. This was observed in both one- and two-dimensional immunoblots. Therefore, the sera tested in this study were additionally screened for reactivity with porcine muscular (α-) and cytoskeletal (β-) actin.

## Results

### Study design and screening of porcine sera for autoreactive antibodies

A collection of sera obtained from experimentally *M. suis*-infected pigs was available from earlier studies [11,13]. The sera were divided into two groups according to their history. Group 1 (36 sera) were derived from pigs (n = 13) that had been vaccinated with MSG1 prior to splenectomy and challenge with *M. suis*. In view of the known major occurrence of autoreactive IgG antibodies during clinical attacks [11], the sera used for the present study were drawn at following time points: I, 21 days post-immunisation and 14 days prior to *M. suis *infection; II, during the first clinical attack (on average 10 days after infection); III, 14 days apart; IV, during the second clinical attack (on average 6 weeks post-infection). Owing to animal deaths, fewer sera were analysed at time points II (n = 11), III (n = 6) and IV (n = 6). Group 2 (40 sera) originated from 10 pigs that had not been immunised prior to splenectomy and *M. suis *infection. For this study, sera taken prior to infection (I), during the first clinical attack (II), between the first and the second clinical attacks (III) and during the second clinical attack (IV) were considered. In total, 76 sera (36 of group 1 and 40 of group 2) were screened by ELISA for reactivity with IgG-depleted antigen preparations from healthy pigs (neg Ag). OD values were normalised to the negative control. The cut-off value was determined to be 0.226 (three times the standard deviation of negative controls).

**Figure 1:**
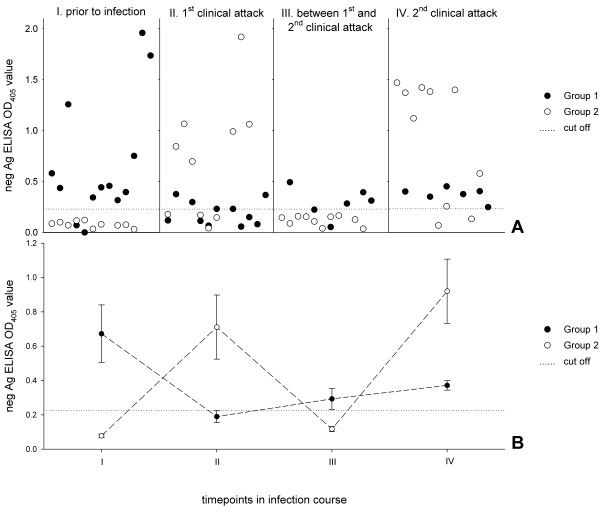
**Serum reactivities with neg Ag at several time points (I to IV)**. A: Group 1 comprises animals (n = 13) that were immunised with MSG1 prior to infection with *M. suis *and splenectomy. Pigs in group 2 (n = 10) were splenectomised and infected with *M. suis*. B: Occurrence of autoreactive antibodies over the time course of infection; mean values for each group are shown. Bars indicate standard errors of the means.

Figure 1A gives an overview of the screening results. At time point I, prior to splenectomy and infection, 84.6% of group 1 sera (immunised with MSG1) and no group 2 sera showed autoreactivity. There was a shift in antibody response during the first clinical attack. The reactivity of group 1 sera decreased to 45.5%, and 60.0% of group 2 sera reacted with neg Ag. Between two clinical attacks, 83.3% of the sera from immunised pigs showed autoreactivity, and antibodies to neg Ag were completely down-regulated in the group 2 sera. During the second clinical attack, 100.0% of sera in group 1 and 80.0% of those in group 2 were autoreactive. Reactivities differed significantly between the groups at every time point. The *P *values were ≤ 0.006, 0.010, 0.004 and 0.043 for I, II, III and IV, respectively. In only one of the 23 animals were there no autoreactive antibodies in sera taken at any time point. Figure 1B shows the occurrence of autoreactive antibodies over the time course of infection in each group. The vaccinated animals representing group 1 had already produced autoreactive antibodies after immunisation with MSG1 and prior to infection and splenectomy, and these antibodies remained at a similar level for time points II, III and IV. The animals of group 2 showed no autoreactive antibodies before infection, up-regulated antibodies to neg Ag at time point II (1^st ^clinical attack), down-regulated antibodies below the cut-off value between clinical attacks, and production of autoreactive antibodies again during the 2^nd ^clinical attack (IV).

### Reactivity with porcine α-actin

**Figure 2:**
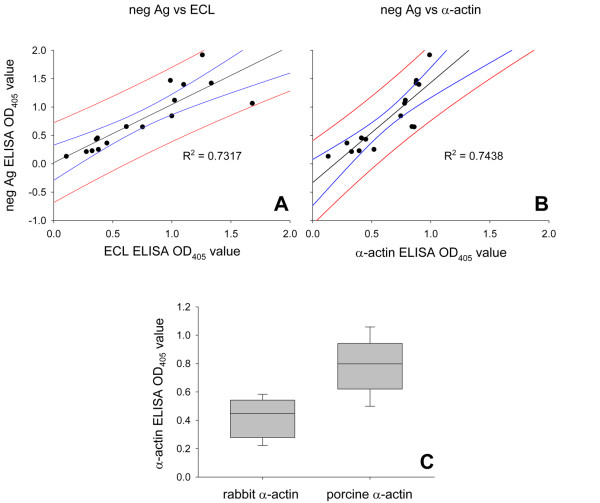
**Actin as autoantigen and species specificity**. Linear regressions between reactivities with neg Ag and ECL (A) and with neg Ag and α-actin (B) are shown for 16 sera. In addition, 95% confidence and 95% prediction bands are shown. C: comparison between reactivities with rabbit and pig α-actin.

In order to identify the autoantigens, the autoreactive sera (n = 36) were further tested with IgG-depleted erythrocyte lysates (ECL) and with porcine α-actin. Earlier studies suggested actin as a potential target of warm autoreactive antibodies (one- and two dimensional immunoblots) [12]. In 32 sera (88.9%), antibodies reactive with both preparations were detected. Linear regression for neg Ag with ECL and α-actin revealed R^2 ^values of 0.732 and 0.744, respectively (Fig. 2).

The actin reactivity was shown to be species-specific, i.e. a significantly lower reactivity was observed with rabbit α-actin than with porcine α-actin. The mean OD values at λ = 405 nm were 0.408 ± 0.136 and 0.793 ± 0.185 for rabbit and porcine α-actin, respectively.

### Reactivity with porcine β-actin

**Figure 3:**
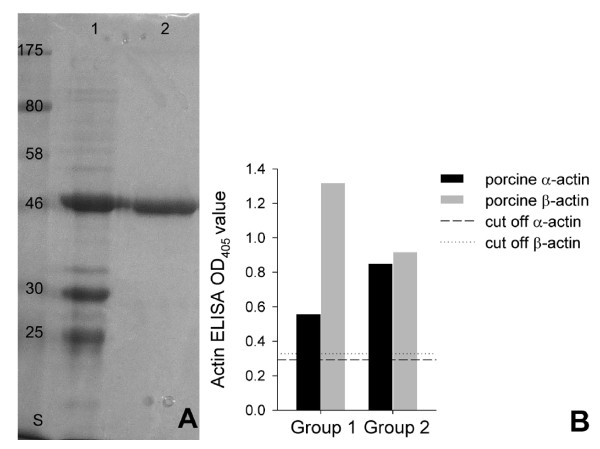
**Purification of porcine β-actin and comparison of serum reactivities with α-actin and β-actin**. A: Coomassie-stained polyacrylamide gel showing purification of recombinant porcine β-actin. Nickel affinity chromatogryphy (1) and electro-elution (2) are shown. Protein mass standards in kDa are indicated in column S; B: Comparison of reactivity with α-actin and β-actin. Group 1, i.e. immunised animals, showed significantly stronger reaction with cytoskeletal actin (*P *≤ 0.0002). No difference between α- and β-actin reactivities was observed in group 2 sera (*P *≤ 0.2034).

The erythrocyte contains cytoskeletal actin (β-actin). This actin form is highly homologous but not identical to muscle actin (α-actin). Since β-actin was not commercially available, the gene was synthetically produced and adapted to *E. coli *codon usage. The protein was expressed, purified and used for the experiments (Fig. 3). Sera reactive with α-actin were tested further with β-actin. They were all positive for both actin forms. Group 1, the MSG1-immunised animals, showed a strikingly stronger reactivity with β-actin than with α-actin (*P *≤ 0.0002). No significant difference was seen in the group 2 animal sera (*P *≤ 0.2034).

### Isotypes of autoreactive antibodies to actin

In warm autoimmune haemolytic anaemia, IgG_1 _and IgG_3 _antibodies predominate [14]. These antibodies are recognised preferentially by macrophages. To gain insight into the autoreactive mechanisms occurring during an *M. suis *infection, secondary antibodies to porcine IgG_1 _and IgG_2 _were used to evaluate the subtypes of actin-reactive antibodies in group 1. No secondary antibodies to porcine IgG_3 _were available. The IgG_1_/IgG_2 _and IgG_2_/IgG_1 _ratios were calculated to be 1.280 ± 1.796 and 2.727 ± 1.925, respectively (*P *≤ 0.018).

### Search for shared epitopes between MSG1 and actin

**Figure 4:**
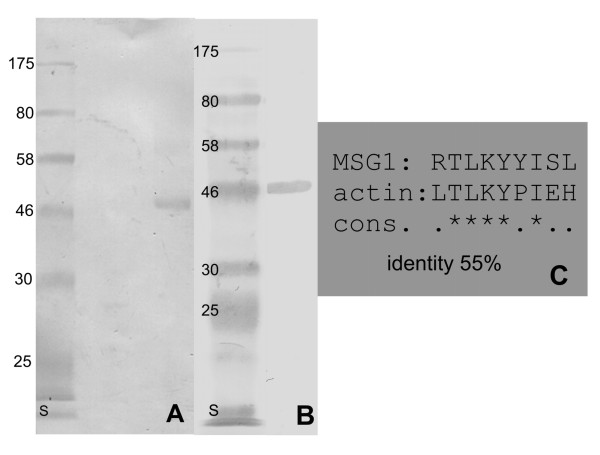
**Cross-reactivity of rabbit hyperimmune sera between actin and MSG1**. A: Westernblot reactivity of MSG1 with a rabbit serum specific for porcine α-actin; B: Porcine actin is detected by a rabbit serum specific for MSG1, column S in A and B indicates protein masses in kDa; C: potential cross-reactive nonapeptides that would be presented by SLA*002 molecule. They share an identity of 55%.

Reactivity with actin was observed in MSG1-vaccinated immunocompetent animals prior to splenectomy and *M. suis *infection. To support the hypothesis of molecular mimicry, we evaluated the reactivity of a rabbit serum targeting recombinant MSG1 with actin and vice versa, i.e. the reactivity of a serum recognising porcine actin with MSG1. Cross-reactivity was observed (Fig. 4). Therefore, the protein sequences of porcine actin and MSG1 were used as input to an epitope finder program. The program allows epitopes potentially presented by SLA molecules to B-cells to be identified with high probability. SLA-2*0201 would present the peptide LTLKYPIEH derived from actin and the peptide RTLKYYISL derived from MSG1. Both peptides are nine amino acids in length and share a sequence identity of 55%. This peptide is identical in both actin forms.

## Discussion

In *M. suis *infections, autoreactive antibodies are of central importance for inducing anaemia [9,10]. Recently we showed that warm autoreactive antibodies of the isotype IgG are up-regulated during the acute phase of experimentally-induced *M. suis *infection [11]. Two-dimensional immunoblot analysis revealed that actin is potentially recognised by these antibodies [11,12]. Actin is known to play a role in autoimmune hepatitis type 1 (AIH-1), where F-actin-reactive antibodies are characteristic [15]. To our knowledge, this is the first report and evidence for autoreactive antibodies directed against actin in warm AIHA. In particular, characterising these autoantigenic structures is an essential basis for understanding the pathogenesis of warm AIHA, whether associated with *M. suis *infections or in general. Typically, autoreactive antibody production is induced by a misguided up-regulation of naturally-occurring B-cells specific for self-antigens, the occurrence of altered self-antigens, the appearance of previously cryptic antigens, and loss of tolerance to self-antigens due to molecular mimicry.

Antibodies recognising cytoskeletal components such as actin, spectrin or band 3 are physiological components of tissue homeostasis and B-cell clones specific for actin patrol continually [16]. A misregulation due to *M. suis *infection could lead to excessive thymus-independent proliferation of these B-cell clones [10]. Other *Mycoplasma *species, e.g. *M. pneumoniae*, are polyclonal B-cell activators of mouse splenocytes [17]. Further, Zachary and Smith have described such misdirected immune responses in *M. suis *infections [10]. Interestingly, actin-targeting IgG antibodies are up-regulated during the first clinical attack when *M. suis *is present in large numbers. This provides evidence for the hypothesis of a direct mitogenic stimulus derived from *M. suis *leading to non-specific B-cell stimulation.

A further explanation for the development of autoreactive antibodies is that damage to red blood cells makes hidden cytoskeletal proteins accessible to circulating antibodies, whereupon they are interpreted as non-self and elicit an immune response. It has been hypothesised that autoimmune epitopes on red blood cells may be result from contact with proteolytic enzymes [18]. Electron microscopic studies have revealed a close association between *M. suis *cells and erythrocytes and striking membrane deformations on the host cells [19,20]. Considering loads of up to 4000 *M. suis *cells per erythrocyte, it is quite evident that the cytoskeleton is modified by attachment and invasion.

The last explanation for the induction of autoreactive antibodies directed against host actin in *M. suis *infections is molecular mimicry. In this process, a peptide derived from a pathogen and presented by MHC II must be capable of activating a self-reactive helper T cell and, therefore, stimulating specific antibody-producing B-cells.

Remarkably, in sera obtained from pigs immunised with MSG1, autoantibodies against actin were already detectable after immunisation but prior to splenectomy and challenge with *M. suis*. These findings strongly support the hypothesis that MSG1 is involved in inducing autoimmunity by molecular mimicry. Evidence for cross-reactivity between MSG1 and actin was found by western blotting using hyperimmune rabbit sera. To test whether actin and *M. suis *actually share potentially cross-reactive epitopes, the NetMHCpan algorithm was used and gave a positive result [21]. Porcine SLA-2*0201 (swine lymphocyte antigen) would strongly bind and therefore present the deduced peptides LTLKYPIEH (from porcine actin) and RTLKYYISL (from MSG1) to the same circulating T-cells. The identity between these peptides was calculated to be 55%, which is enough for cross-reactivity. Degeneracy in both the TCR and MHC peptide-binding motifs reduces the sequence-specific requirement to only a few crucial residues [22-24]. It remains to be elucidated by systematic *in vitro *stimulation assays whether these peptides actually play a role in the pathogenesis of AIHA due to *M. suis *infection.

In RBCs and many other cell types β-actin is part of the cytoskeleton; α-actin predominates only in muscle cells. If the strongly β-actin-binding antibodies identified encounter their target ubiquitously, RBCs would not be the only cells affected. There is evidence that lymphocytes and epithelial cells are harmed during *M. suis *infection (unpublished observations). Cytoskeletal proteins in cells with intact plasma membranes are cryptic for antibodies. However, if the cell is injured by interaction with *M. suis*, β-actin would become accessible. Antibodies recognising this actin opsonise the red blood cells which leads to their removal from the circulatory system and therefore to anaemia. Notably, clinical signs expected by autoreactive processes targeting muscle actin (α-actin) were observed.

In autoimmune diseases including AIHA, misdirected IgG_1 _and IgG_3 _antibodies play an important role because they bind efficiently to FcReceptorIII (FcRIII) molecules on macrophages [14,25]. Interestingly, the antibodies that predominantly recognised actin were of the subtype IgG_2_rather than IgG_1_. However, a definitive subtype assignment was not feasible owing to high standard deviations. Immune complexes with the IgG_2 _subtype activate the alternative complement cascade pathway and are important in defence against encapsulated bacteria [26,27]. However, the consequences of autoreactive IgG_2 _up-regulation during *M. suis *infection remain unclear. Unfortunately, no statement about the presence of IgG_3 _antibodies and their role in *M. suis *infection can be given since secondary antibodies for this porcine subtype were not available.

## Conclusions

Overall, we record that autoreactive IgG antibodies up-regulated during infectious porcine anaemia induced by *M. suis *recognise actin. These autoreactive antibodies are obviously involved in the pathogenesis of the severe anaemia observed during the infection. An interplay of several mechanisms is assumed, i.e. a blastogenic response due to unspecific lymphocyte proliferation, direct damage to red blood cells and molecular mimicry. To our knowledge, this is the first report of autoreactive actin antibodies in relation to AIHA. In our opinion, the experimentally *M. suis*-infected splenectomised pig represents a useful model for investigating AIHA because of the controllable inducible symptoms.

## Methods

### Preparation of screening antigens

To screen for autoreactive antibodies, blood from healthy pigs was prepared as previously described for *M. suis *antigen preparations [28,29] with slight modifications. Briefly, blood cells from *M. suis*-negative pigs were sedimented by centrifugation at 300 × g for 15 min at room temperature (RT). Plasma and buffy coat were discarded. The erythrocytes were suspended in phosphate-buffered saline (1 × PBS; Biochrom Ag, Berlin, Germany) containing 0.15% Tween20 and 3% EDTA and incubated for 20 min at RT with shaking. Debris and erythrocytes were removed by low-speed centrifugation (500 × g, 20 min, RT). The supernatant was centrifuged at 25,000 × g for 120 min at 4°C. The resulting pellet was resuspended (1 mg/ml) in sterile 1 × PBS. The preparation was depleted of IgG and albumin using a ProteoExtract^® ^Albumin/IgG Removal Kit, Maxi (Calbiochem, Merck, Geneva, Switzerland) according to the manufacturer's recommendations and stored in 100 μl aliquots at -80°C until further use. The preparations were referred to as negative antigen (neg Ag).

Sera reactive with neg Ag were further tested with IgG-depleted erythrocyte lysate (ECL) and with several actin preparations. ECL was prepared by hypotonic lysis of erythrocytes from a healthy pig. Red blood cells were washed three times with 1 × PBS (500 × g, 15 min, RT) and resuspended in ice cold lysis buffer (5 mM Na_2_PO_4_, 1 mM EDTA, pH 7.6, Sigma) at a 1:1 (v/v) ratio. After incubation on ice for 5 min, the ghosts (remnants of lysed cells) were pelleted (30,000 × g, 15 min, 4°C). This procedure was repeated until the pellet turned white. The white pellet was washed in 1 × PBS, homogenised by ultrasonication on ice and IgG depleted. The concentration was set to 1 mg/ml with 1 × PBS and the suspension was stored in 1 ml aliquots at -80°C until further use.

Pig and rabbit muscle actin (α-actin) were obtained from Sigma, Buchs, Switzerland. Porcine cytoskeletal actin (β-actin) was not commercially available and was therefore produced recombinantly (see below).

### Screening of sera by ELISA

Sera were obtained in previous studies using a splenectomized pig model for experimental *M. suis *infections [13]. They were divided into two groups. Group 1 consisted of sera from animals that had been immunised with a recombinant immunogenic surface protein of *M. suis *(MSG1) [13] prior to splenectomy and *M. suis *infection. Group 2 sera were from splenectomised, *M. suis*-infected pigs that had not been immunised [11]. The studies were approved by the government of upper Bavaria under the registration number 211-2531-77/98 and were performed in compliance with animal care legal prescriptions.

The porcine sera were screened for autoreactivity by ELISA as described previously [30]. Briefly, 100 ng of IgG-depleted neg Ag were coated on individual wells of microtiter plates. Sera were diluted 1:100 in 1 × PBS containing 0.05% (v/v) Tween20 (PBST) and tested in duplicate. Sera were drawn prior to *M. suis *infection, after immunisation in group 1 (I), during the first clinical attack (II), between first and second clinical attacks (III), and during the second clinical attack (IV). Sera reactive with neg Ag were further tested for reactivity with ECL, pig and rabbit α-actin, and porcine β-actin. The protein concentration was 100 ng/well. OD values (λ = 405 nm) were determined and normalised to a negative control (pool of 10 sera from healthy pigs). Cut-off values were set at three times the standard deviation of the negative controls.

To determine the subtypes of autoreactive antibodies, monoclonal antibodies targeting porcine IgG_1 _and IgG_2 _(Prionics, Schlieren, Switzerland) were diluted 1:1000 in PBST. Horse-radish-peroxidase-labelled antibodies against mouse IgG (Sigma) diluted 1:5000 in PBST were used for detection.

### Cloning and expression of porcine cytoskeletal actin

Porcine cytoskeletal actin (β-actin) was not commercially available. Using the encoding mRNA sequence (Genbank:AY550069), the gene was *de novo *synthesised and optimised for *E. coli *codon usage by Eurofins (Martinsried, Germany). For cloning, specific recognition sites for endonucleases were introduced (*Xho*I at the 5' end and *Hind*III at the 3' end). For expression, the gene was ligated into the expression vector pBadMycHisA (Invitrogen) using T4 ligase from Roche, Basel, Switzerland (overnight, 14°C). The insert-containing vector was transformed into *E. coli *LMG194 (Invitrogen). The *E. coli *transformants were grown at 37°C, and expression of the recombinant porcine β-actin was induced by adding 0.02% arabinose at OD_600 _= 0.4. The cells were incubated for a further 4 h at 37°C and the 6×His-tagged protein was purified by nickel affinity chromatography (GE Healthcare, Glattbrugg, Switzerland) according to the manufacturer's recommendations. To extract the protein from inclusion bodies, 8 M urea was used for purification. The recombinant protein was further purified by electro-elution from 10% polyacrylamide gels using an Electro-Eluter Model 422 (Biorad, Reinach, Switzerland). The eluted protein was precipitated by adding three volumes of ice cold acetone and incubating overnight at -20°C. The protein was harvested by centrifugation (10,000 × g, 90 min, 4°C), allowed to dry, resuspended in 1 × PBS, and stored in aliquots of 1 mg/ml at -80°C.

### Production of hyperimmune sera

One milligram of protein (recombinant MSG1 [13], recombinant porcine β-actin or porcine α-actin (Sigma, Buchs, Switzerland)) was mixed with complete Freund's adjuvant (Sigma) at a 1:1 (v/v) ratio and injected subcutaneously into rabbits. Two booster injections (the proteins were mixed 1:1 (v/v) with incomplete Freund's adjuvant) were given two and four weeks later. Six weeks after the first injection the rabbits were bled; the serum was harvested and stored in aliquots at -20°C. Immunisations were approved by the Veterinary Office of Zurich and conducted under the registration number 144/2008 in accordance with legal prescriptions.

### Western blotting

The cross-reactivity of rabbit sera against MSG1 and porcine α-actin between α-actin and MSG1 was tested by western blotting as described previously [11]. Briefly, α-actin and MSG1 were loaded on 10% polyacrylamide gels containing 1% SDS (w/v) and transferred to a nitrocellulose membrane (Whatman Protran^®^, GE Healthcare). Free binding positions on the membrane were blocked with skim milk (Sigma, 2% w/v in Tris buffered saline, TBS) and probed with rabbit hyperimmune sera (diluted 1:100 in TBS containing 2% skim milk powder) against recombinant MSG1 and α-actin, respectively. A secondary antibody reactive with pig IgG (Sigma) and coupled to horseradish peroxidase (HRP) was used, diluted 1:5000 in TBS containing 2% (w/v) skim milk powder. The substrate for the HRP was H_2_O_2 _(Sigma); 4-Chloro-1-naphthol (Sigma) was used as chromogen. The reaction was stopped by adding MQ water (Millipore, Zug, Switzerland).

### Search for cross-reactive epitopes

To identify potential cross-reactive epitopes, an epitope finder program referred to as the NetMHCpan algorithm was used [21]. Published protein sequences of MSG1 (UniProtKB/TrEMBL:Q05G10), porcine α-actin (Swiss-Prot:P68137.1) and porcine β-actin (Swiss-Prot:Q6QAQ1.2) were used as input.

### Statistical analysis

The ELISA OD values measured at 405 nm were compared by linear regression models using SigmaPlot software, Version 10.0 (SPSS Inc., Chicago, IL, U.S.A). To show independence of values between the two groups, the unpaired Student's *t-*test was applied (significant difference if *P *≤ 0.05).

## Authors' contributions

KMF performed the experiments with group 1 sera and drafted the manuscript; KH performed the experiments with group 2 sera and helped to draft the manuscript; K Heinritzi designed the group 2 animal experiments and performed them; the animal experiments of group 1 were designed and organised by MR; LH designed, planned and coordinated the study overall, supervised the experiments and helped to draft the manuscript. All authors read and approved the final manuscript.
